# Predictive Factors of Surgical Recurrence in Patients with Crohn’s Disease on Long-Term Follow-Up: A Focus on Histology

**DOI:** 10.3390/jcm11175043

**Published:** 2022-08-27

**Authors:** Gian Paolo Caviglia, Chiara Angela Mineo, Chiara Rosso, Angelo Armandi, Marco Astegiano, Gabriella Canavese, Andrea Resegotti, Giorgio Maria Saracco, Davide Giuseppe Ribaldone

**Affiliations:** 1 Department of Medical Sciences, University of Turin, 10126 Turin, Italy; 2 Division of Gastroenterology, AOU Città della Salute e della Scienza–Molinette Hospital, 10126 Turin, Italy; 3 General Surgery 1U, Città della Salute e della Scienza–Molinette Hospital, 10126 Turin, Italy; 4 Department of Pathology, AOU Città della Salute e della Scienza–Molinette Hospital, 10126 Turin, Italy

**Keywords:** cryptitis, granulomas, inflammatory bowel diseases, IBD

## Abstract

In patients with Crohn’s disease (CD) that underwent surgery, predictive factors of surgical recurrence have been only partially identified. The aim of our study was to identify potential factors associated with an increased risk of surgical recurrence. A monocentric retrospective observational study was conducted including patients diagnosed with CD, according to ECCO criteria who received their first ileocolic resection. Overall, 162 patients were enrolled in our study; 54 of them were excluded due to a lack of sufficient data. The median follow-up was 136.5 months, IQR 91.5–176.5, and the surgical recurrence rate after the median follow-up was 21.3%. In the multivariate analysis, an age ≤ 28 years at the first surgical resection (aHR = 16.44, *p* < 0.001), current smoking (aHR = 15.84, *p* < 0.001), female sex (aHR = 7.58, *p* < 0.001), presence of granulomas at local lymph nodes (aHR = 12.19, *p* < 0.001), and treatment with systemic corticosteroids after the first surgical resection (aHR = 7.52, *p* = 0.002) were factors significantly associated with a risk of surgical recurrence, while cryptitis resulted in a protective factor (aHR = 0.02, *p* < 0.001). In conclusion, the heterogeneous spectrum of factors associated to the risk of surgical recurrence in patients with CD that underwent ileocolic resection supports the need of a personalized follow-up taking into account different clinical, surgical, and histologic features.

## 1. Introduction

Crohn’s disease (CD) is a chronic inflammatory disorder that can affect any segment of the gastrointestinal tract, from the mouth to the anus [1]. The etiopathogenesis of CD is still largely unknown; likely, the disease is the result of the interaction between genetic susceptibility, environmental factors, and intestinal microbiome, which lead to an abnormal mucosal immune response and to an impairment of the intestinal barrier function [2,3,4]. 

Despite recent advances in the medical therapy of CD, approximately 70% patients will undergo surgery, and more than 20% of them will experience surgical recurrence after 10 years [5,6]. Worthy of mention is the risk of developing short bowel syndrome in patients with CD undergoing multiple resections; approximately 60% of CD patients with short bowel syndrome are permanently dependent on parenteral nutrition [7].

An early study published in 1981 already reported a 50% reoperation rate in 146 patients with CD at 14 years after first surgery [8]. Subsequently, in a larger cohort of 639 patients that required surgical intervention for their CD, a recurrence rate of 34% at 10 years has been reported; the number of intestinal sites involved was associated with the intra-abdominal recurrence rate, while the perineal disease was associated with the risk of local recurrence [9]. Though recurrence usually affects the site of the original surgery, one-third of recurrences occur elsewhere in the bowel according to the intervention site and operative technique [10].

To date, several studies investigated factors associated with surgical recurrence, mainly focusing on clinical and surgical features. Despite being controversial, data such as family history, smoking habit, age at surgery, type of surgical intervention, and subsequent type of medication have been suggested as risk factor for reoperation [11,12,13,14]. However, to the best of our knowledge, data on the association between histopathological features and the risk of surgical recurrence are scanty. 

The purpose of our study was to identify potential predictive factors, related to the clinical characteristics of the patient, the characteristics of the disease, the anatomopathological characteristics of the resected intestinal tract and the surgical technique, associated with an increased risk of long-term surgical recurrence in patients with CD that underwent ileocolic resection.

## 2. Materials and Methods

### 2.1. Patients

In this single-center study, we retrospectively enrolled patients with CD in follow-up (FU) at the outpatients clinic of the Unit of Gastroenterology of “Città della Salute e della Scienza di Torino–Molinette” Hospital, Turin, Italy, that underwent first ileocolic resection between 2000 and 2013.

The study inclusion criteria were: diagnosis of CD according to the criteria of the European Crohn’s and Colitis Organization (ECCO) [15,16] and first surgery for right ileo-colic resection performed at the General Surgery of the “Città della Salute e della Scienza di Torino–Molinette” Hospital. Exclusion criteria were: lack of data on post-surgery FU, lack of data concerning anatomopathological examination, lack of data on type of surgical procedure, and surgery other than right ileo-colic resection.

For all patients included, we collected the following data: age, sex, smoking habit, family history of IBD, clinical history (i.e., age at diagnosis, age at first resection, disease location, and behavior), medical therapy administered after the first surgery (i.e., mesalamine, thiopurine, systemic steroids, and biologics), features related to the surgical intervention (i.e., length of bowel resection, type of surgical intervention, type of anastomosis performed, and temporary or permanent ostomy), and anatomopathological characteristics (i.e., stenosis, fistulas, pseudopiloric metaplasia, basal plasmacytosis, granulomas at loco-regional lymph nodes, surgical margin, degree of inflammation, hyper-eosinophilia, colonic microscopic inflammation, reactive lymphoid hyperplasia, cryptitis, serositis, perivisceritis, and inflammatory pseudopolyps). Hematoxylin eosin saffron stain was applied to full-thickness 3-mm sections of paraffin blocks of the ileal border. An experienced pathologist examined each part (G. C.). To evaluate CD-related lesions affecting each layer of the intestinal wall, an analytical grid was created (mucosa, submucosa, and subserosa or muscularis). We measured the length of the ileal resection for each patient (in centimeters) and the separation between the ileal margin and the first CD mucosal ulceration seen by the pathologist on the opened material for macroscopic inspection (in centimeters). If CD’s mucosal ulcerations were seen on the ileal margin, the margin was considered to be “macroscopically impacted”.

### 2.2. Statistical Analysis

All data were collected in a dedicated Microsoft Excel^®^ database. Statistical analysis was performed using MedCalc Statistical Software version 18.9.1 (MedCalc Software bvba, Ostend, Belgium).

The distribution of continuous variables was checked by D’agostino–Perason test (*p* < 0.05 = reject normality). According to data normality, continuous variables were reported as mean ± standard deviation (SD) or as median and interquartile range (IQR). Categorical variables were expressed as frequencies (*n*) and percentage (%). Survival curves were calculated according to the Kaplan–Meier method; differences between survival curves were assessed by the Log-rank test. Univariate and multivariate analysis for variables associated to the risk of surgical recurrence was performed by Cox proportional-hazard regression; the strength of association was reported as Hazard Ratio (HR) with the corresponding 95% confidence interval (CI). In order to check for potential interaction among variables, all variables were tested at multivariate Cox regression, irrespectively from the statistical significance at univariate analysis; by a backward approach, we first entered all variables into the model and next removed the non-significant variables sequentially.

For all analyses, a *p* value < 0.05 was considered statistically significant.

## 3. Results

A total of 162 medical records from patients with CD that underwent ileocolic resection between 2000 and 2013 were evaluated. Overall, 54 patients were excluded due to the lack of data (*n* = 52) or lost to FU (*n* = 2). Of the 108 patients included in the study, 36 of them had at least one surgical recurrence (33.3%); the baseline characteristics are reported in Table 1.

**Figure 1:**
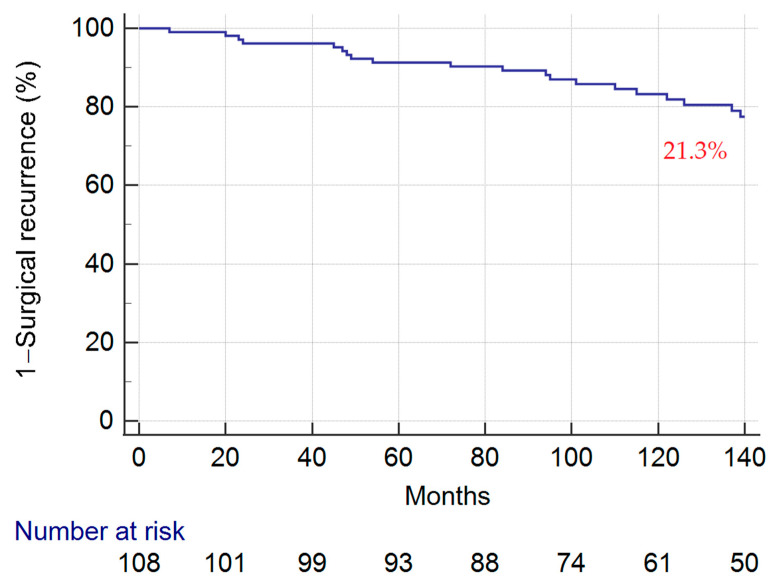
Cumulative surgical recurrence rates calculated using the Kaplan–Meier method.

The median FU was 136.5 (91.5–176.5) months; at 137 months, 23 (21.3%) patients had surgical recurrence (Figure 1). To note, among the thirty-six patients with surgical recurrence, ten (27.8%) experienced more than one recurrence: six (16.7%) patients underwent two ileocolic resections, two (5.6%) underwent three ileocolic resections, and two (5.6%) patients underwent four ileocolic resections. 

**Figure 2:**
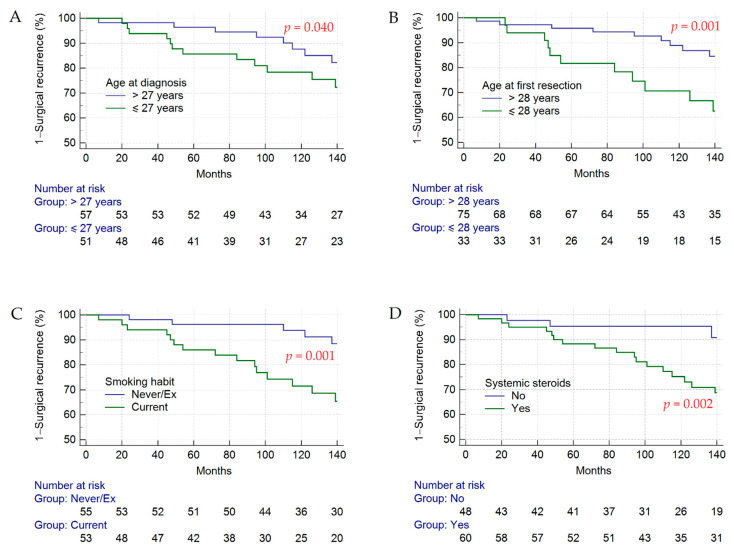
Cumulative surgical recurrence rates calculated using the Kaplan–Meier method. At the median FU of 136.5 months, the 82.2% of patients with age > 27 years at CD diagnosis was surgical recurrence-free, compared to the 75.5% of patients with age ≤ 27 years at CD diagnosis (**A**), the 84.6% of patients with age > 28 years at first surgery was surgical recurrence-free as compared to the 66.7% of patients with age ≤ 28 years at first surgery (**B**), the 90.1% of former/never smokers was surgical recurrence-free as compared to the 68.7% of current smokers (**C**), and the 92.7% of patients that did not receive systemic steroid therapy was surgical recurrence-free as compared to the 70.9% of patients administered with systemic steroid therapy (**D**).

In the univariate analysis, we observed different cumulative incidences of surgical recurrence according to age at CD diagnosis ≤ 27 years (Log-rank test, *p* = 0.040), age at first surgery ≤ 28 years (Log-rank test, *p* = 0.001), current smoking status (Log-rank test, *p* = 0.001), and administration of systemic steroids after surgery (Log-rank test, *p* = 0.002) (Figure 2). Interestingly, in the multivariate Cox regression analysis, we observed that an age ≤ 28 years at the first surgical resection (aHR = 16.44, *p* < 0.001), current smoking (aHR = 15.84, *p* < 0.001), female sex (aHR = 7.58, *p* < 0.001), cryptitis (aHR = 0.02, *p* < 0.001), presence of granulomas at the local lymph nodes (aHR = 12.19, *p* < 0.001), and treatment with systemic corticosteroids after the first surgical resection (aHR = 7.52, *p* = 0.002) were factors significantly associated with the risk of surgical recurrence (Table 2).

## 4. Discussion

In the present study, we investigated the surgical recurrence rate in patients with CD and potential predictors of surgical recurrence. In agreement with the literature data, we observed that the 21.3% of our patients had surgical recurrence during a median FU of 136.5 months; the age at first surgery, female sex, smoking habit, cryptitis, presence of granulomas at loco-regional lymph nodes, and systemic corticosteroid administration after the first surgery resulted in independent predictors of surgical recurrence. As a matter of fact, our results are somehow consistent with those reported in a seminal study published in 2006, where the authors observed that the requirement for steroid use, an age < 40 years at CD diagnosis, and the presence of a perianal disease were all independent factors associated with a disabling disease course, whose definition included intestinal resection [17].

Among patients-related variables, a smoking habit is a well-known risk factor associated to CD development, with a four-fold higher risk of CD development in current smokers in comparison to subjects that had never smoked [18]. Here, we observed that current smoking was one of the strongest predictors of surgical recurrence; our results agree with the study of Unkart and colleagues [11], that reported a higher risk of second ileo-colectomy for current smokers at the time of first surgical intervention (HR = 2.08, *p* = 0.023). Taken together, these results highlight that smoking not only has a significant impact on CD development, but also on the progression of the disease, increasing the risk of further surgery. Additionally, a younger age at first surgery, probably as a surrogate of a more aggressive disease, was associated with an increased risk of surgical recurrence; our results agree with the study of Wang and colleagues where it was reported that an early age at first CD surgery (OR = 1.12, *p* < 0.001) predicted a higher surgical recurrence risk [19]. Finally, we found that the female sex was associated to surgical recurrence; to date, sex has been evaluated in several studies, but most found no difference between the sexes [20].

Among histology-related factors, we observed that cryptitis was associated with a reduced risk of surgical recurrence. A previous French study investigated the association between histologic features of the ileal margin with disease recurrence in a cohort of 211 patients with ileal or ileocolonic CD; contrarily to our findings, the authors pointed out that transmural lesions were associated with an increased risk of post-operative recurrence (endoscopic recurrence, OR = 3.83, *p* = 0.008; clinical recurrence, OR = 2.04, *p* = 0.026) [21]. Together with the paucity of the available evidence, it is likely that the differences in the clinical characteristics among the study cohorts and the different study endpoints could have led to conflicting results. Further studies are needed to better elucidate this aspect. Concerning the presence of intestinal granulomas in tissue specimens, a 2010 meta-analysis of 22 studies (2236 patients) reported an association between granulomatous CD and an increased risk of recurrence and reoperation [22]. In addition, a subsequent study found that the presence of granulomas in the mesenteric lymph node was a significant risk factor for both postoperative endoscopic (*p* = 0.015) and surgical recurrence (*p* = 0.035) [23]. These results are consistent with our findings, where the presence of granulomas at loco-regional lymph nodes was independently associated with an increased risk of surgical recurrence (aHR = 12.19, *p* < 0.001). Furthermore, the presence of granulomas in resection specimens has been recognized as a risk factor for post-operative recurrence by the ECCO guidelines, together with smoking, the absence of prophylactic treatment, a penetrating disease at index surgery, a perianal location, and myenteric plexitis [24]. However, it must be acknowledged that the definition of post-operative recurrence varies greatly among studies. Indeed, CD recurrence could be considered clinical, endoscopic, radiological or surgical; our study was focused on surgical recurrence only, thus contributing to explain the discrepancies between our results and those from previous studies.

Finally, concerning medications, we observed that patients treated with systemic corticosteroids after surgery were those with a higher incidence of surgical recurrence (aHR = 7.52, *p* = 0.002). This result could be explained by the fact that patients receiving systemic corticosteroids had a more aggressive post-surgical disease phenotype, thus being intrinsically at risk of disease progression, including surgical recurrence.

Our study has some limitations that should be acknowledged. Firstly, given the retrospective nature of the study, we cannot rule out possible biases related to data loss; furthermore, the wide observation period (2000–2013) may have led to non-homogeneous data collection over time. In addition, data on extraintestinal manifestations were not systematically available for all patients; though CD patients with liver involvement (such as primary sclerosing cholangitis) could have a phenotypical and clinical pattern that sharply differs from patients with CD alone [25]. For the abovementioned reason, we were not able to investigate this aspect in our series. Secondly, the number of patients included in the study was relatively small, which may have had an impact on the lack of statistical significance for variables such as perianal diseases and the presence of fistulas, which have been consistently identified as risk factors for surgical recurrence by previous studies. Lastly, the single-center study design may represent an additional limitation. Nevertheless, all patients included in the study were followed with regular scheduling by the same gastroenterologist (M.A.), surgical procedures were performed by the same surgeon (A.R.), and histologic examination was performed by the same pathologist (G.C.), thus allowing to avoid any potential operator-dependent bias. 

## 5. Conclusions

In conclusion, we observed a heterogeneous spectrum of factors associated to the risk of surgical recurrence in CD patients that underwent ileocolic resection. In particular, other than patient- and surgical-related factors, the presence of granulomas in loco-regional lymph nodes could represent an additional risk factor for surgical recurrence. In patients with CD with past surgical history, a personalized follow-up taking into account different clinical, surgical, and histologic features is mandatory to identify patients at the highest risk of surgical recurrence. 

## Figures and Tables

**Table 1:** Baseline characteristics of the patients included in the study.

Characteristics	*n* = 108
Age at first surgery (years), mean ± SD	38.8 ± 13.8
Age at CD diagnosis (years), median (IQR)	28.5 (22.0–45.0)
Sex (M/F), *n*	60/48
Smoking habit (current/ex/never), *n* (%)	54 (50.0%)/22 (20.4%)/32 (29.6%)
Family history of CD, *n* (%)	20 (18.5%)
Colonic involvement, *n* (%)	54 (50.0%)
Upper gastrointestinal tract involvement (L4), *n* (%) ^1^	13 (12.0%)

^1^ L4, any location proximal to the terminal ileum, except the mouth. Abbreviations: Crohn’s disease (CD), interquartile range (IQR), female (F), male (M), number (*n*), standard deviation (SD).

**Table 2:** Univariate and multivariate Cox regression analysis of factors associated to surgical recurrence.

Variables	Univariate (HR; 95% CI)	*p* Value	Multivariate * (aHR; 95%CI)	*p* Value
Age at first surgery ≤ 28 years	2.95 (1.52–5.76)	0.001	16.44 (4.63–58.34)	<0.001
Age at CD diagnosis ≤ 27 years	1.98 (1.01–3.90)	0.048	/	/
Female sex	1.24 (0.64–2.40)	0.530	7.58 (2.50–22.99)	<0.001
Current smoker	3.28 (1.59–6.78)	0.001	15.84 (4.80–52.23)	<0.001
Family history of CD	0.84 (0.36–1.96)	0.840	/	/
Colonic involvement	0.57 (0.29–1.12)	0.100	/	/
Upper gastrointestinal tract involvement (L4)	1.63 (0.63–4.27)	0.310	/	/
Perianal lesions	1.29 (0.60–2.76)	0.510	/	/
Fistulas	0.68 (0.34–1.34)	0.260	/	/
Stenosis	0.70 (0.29–1.72)	0.440	/	/
Laparoscopic resection	1.14 (0.27–4.78)	0.860	/	/
Length of bowel resection (cm)	1.01 (0.99–1.02)	0.500	/	/
Surgical margins involvement	1.11 (0.49–2.50)	0.800	/	/
Temporary ostomy	0.52 (0.20–1.34)	0.180	/	/
Cryptitis	0.49 (0.17–1.38)	0.180	0.02 (0.00–0.11)	<0.001
Plexitis	2.06 (0.62–6.82)	0.240	/	/
Sierositis	0.97 (0.46–2.02)	0.930	/	/
Perivisceritis	0.23 (0.03–1.68)	0.150	/	/
Colonic microscopic inflammation	0.56 (0.28–1.13)	0.110	/	/
Granulomas at loco-regional lymph nodes	2.16 (0.98–4.77)	0.056	12.19 (3.27–45.46)	<0.001
Reactive lymphoid hyperplasia	1.44 (0.69–3.00)	0.330	/	/
Hyper-eosinophilia	2.68 (0.36–19.90)	0.330	/	/
Pseudopiloric metaplasia	0.23 (0.03–1.68)	0.150	/	/
Inflammatory pseudopolyps	0.64 (0.19–2.09)	0.460	/	/
Anti-TNF administration	0.63 (0.29–1.36)	0.240	/	/
Thiopurine administration	0.55 (0.27–1.12)	0.100	/	/
Corticosteroid administration	4.07 (1.57–10.55)	0.002	7.52 (2.15–26.22)	0.002

* Multivariate Cox regression analysis was performed with a backward approach. Abbreviations: adjusted hazard ratio (aHR), Crohn’s disease (CD), confidence interval (CI), hazard ratio (HR), tumor necrosis factor (TNF).

## Data Availability

The data presented in this study are available upon request from the corresponding author.
